# Triclosan Lacks (Anti-)Estrogenic Effects in Zebrafish Cells but Modulates Estrogen Response in Zebrafish Embryos

**DOI:** 10.3390/ijms19041175

**Published:** 2018-04-12

**Authors:** Hélène Serra, François Brion, Jean-Marc Porcher, Hélène Budzinski, Selim Aït-Aïssa

**Affiliations:** 1 Institut National de l’environnement Industriel et des Risques (INERIS), Unité d’Ecotoxicologie in vitro et in vivo, UMR-I SEBIO 02, BP 2, 60550 Verneuil-en-Halatte, France; serra.helene@gmail.com (H.S.); francois.brion@ineris.fr (F.B.); jean-marc.porcher@ineris.fr (J.-M.P.); 2 Université de Bordeaux, EPOC-UMR 5805, Laboratoire de Physico- et Toxico-Chimie de l’environnement (LPTC), 33405 Talence, France; helene.budzinski@u-bordeaux.fr

**Keywords:** in vitro, in vivo, estrogen receptor, zebrafish, triclosan, brain aromatase

## Abstract

Triclosan (TCS), an antimicrobial agent widely found in the aquatic environment, is suspected to act as an endocrine disrupting compound, however mechanistic information is lacking in regards to aquatic species. This study assessed the ability of TCS to interfere with estrogen receptor (ER) transcriptional activity, in zebrafish-specific in vitro and in vivo reporter gene assays. We report that TCS exhibits a lack of either agonistic or antagonistic effects on a panel of ER-expressing zebrafish (ZELH-zfERα and -zfERβ) and human (MELN) cell lines. At the organism level, TCS at concentrations of up to 0.3 µM had no effect on ER-regulated brain aromatase gene expression in transgenic cyp19a1b-GFP zebrafish embryos. At a concentration of 1 µM, TCS interfered with the E2 response in an ambivalent manner by potentializing a low E2 response (0.625 nM), but decreasing a high E2 response (10 nM). Altogether, our study suggests that while modulation of ER-regulated genes by TCS may occur in zebrafish, it does so irrespective of a direct binding and activation of zfERs.

## 1. Introduction

Triclosan (TCS) is a chlorinated phenolic chemical used as a wide spectrum antimicrobial agent in many personal care products, such as cosmetics. Around 400 tons of TCS was used in Europe in 2007, reflecting its extensive application over the last few decades [1]. As a household chemical, TCS enters the aquatic environment mainly through wastewater treatment plant (WWTP) effluent releases, after incomplete removal by adsorption from sewage sludge [2]. TCS is commonly detected in WWTP effluents [3] and in river water, where it was reported to occur at concentrations ranging from the ng/L range up to 2.3 µg/L [4,5,6].

TCS toxicity for mammalian and several aquatic species has been well documented, as thoroughly reviewed by Dann and Hontela [2]. Among its different potential effects, concern has been raised regarding its endocrine-disrupting activity in aquatic species. TCS was reported to alter endocrine-regulated processes, such as thyroid hormone homeostasis and reproduction. In fish, different outcomes were reported. Foran et al. (2000) reported a sex ratio weakly biased toward males after exposure of medaka fry to 100 µg/L, however not at lower or higher concentrations, suggesting that TCS has a slight androgenic effect [7]. The induction of vitellogenin (VTG) expression in male mosquitofish [8] and medaka [9] was observed after exposure to 100 µg/L TCS, while it did not alter VTG in male fathead minnow [6]. A recent study using medaka reported dual effects, as TCS was shown to decrease VTG expression in males and increase it in females after exposure to concentrations of 174 µg/L and above of TCS [10]. Overall, studies on aquatic vertebrates suggest that TCS has an endocrine-disrupting capacity, however the mechanisms by which it interferes with sex steroid-regulated pathways remain unclear [2].

Several in vitro studies using mammalian cell systems have addressed the ability of TCS to interfere with the human estrogen receptor (hER), with contrasting outcomes. For instance, in one study, TCS was found to weakly transactivate hERα in transiently transfected CV-1 cells [11], and to promote MCF-7 cell growth up to 80% of positive control at 75 µM [12]. In other studies, TCS was reported to lack agonistic effects in similar human ER- and androgen receptor (AR)-dependent reporter gene assays [13,14,15], but to have ER and AR antagonistic activity [13]. To date, most in vitro studies on the anti-estrogenic effects of TCS originate from mammalian test systems, and only a few mechanistic studies have focused on ER signaling pathways in fish. A recent study examined the ability of TCS to activate the ERα ligand-binding domain of different fish species using a transient reporter gene assay in human cells, and showed only weak activation at high µM concentrations of TCS on some fish ERα, but not on others [16]. Despite information obtained so far suggesting the involvement of ER signaling in TCS response, the underlying mechanism of its estrogen-related effects remains unclear, notably in aquatic species.

In this context, the aim of the present study was to assess TCS interaction with ER signaling pathways in a model fish species, the zebrafish (zf), by using recently established in vitro and in vivo zf-specific reporter gene assays. Integrative strategy based on complementary screening bioassays has been shown to be relevant for characterizing the estrogenic potency of chemicals [17], and quantifying estrogenic activity in the environmental matrices [18] in fish. In the present study, we used a panel of in vitro assays based on (1) zf liver cell lines stably transfected to express zfERα (ZELHα), zfERβ1 (ZELHβ1) or zfERβ2 (ZELHβ2) [19]; (2) human breast cancer MCF-7 cells expressing endogenous hERα (MELN) [20]; and an in vivo assay based on (3) transgenic cyp19a1b-GFP zebrafish embryos, which express *cyp19a1b* gene under the strict regulation of ER in radial glial cells (RGCs) [21]. The in vitro and in vivo effect of serial concentrations of TCS was assessed in the presence or absence of the endogenous steroidal estrogen 17β-estradiol (E2), in order to investigate agonistic and antagonistic activity.

## 2. Results

### 2.1. Triclosan Does Not Alter ZfER Transactivation In Vitro

**Figure 1:**
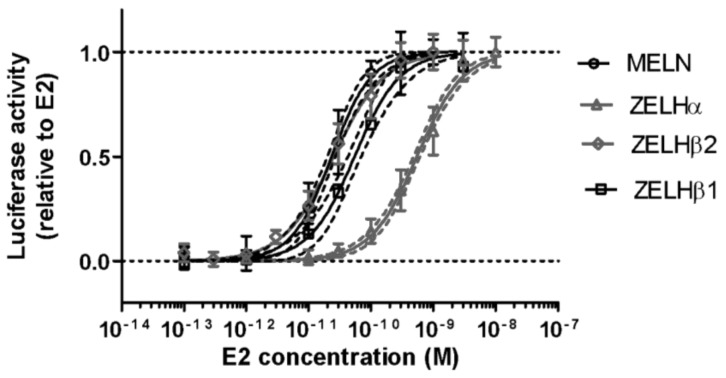
Luciferase induction by E2 in human (MELN) and zebrafish (ZELHα, ZELHβ1, and ZELHβ2) cell lines. Data were normalized to solvent control and to E2 maximal effect. Data are mean values ±SD (technical triplicates) and Hill fitting curve with 95% confidence interval belt.

The different reporter cell lines responded to E2, the reference compound, in the expected range of sensitivity (Figure 1), with EC50s ranging from 0.01–0.02 nM in MELN, ZELHβ1, and ZELHβ2 cells, to 2 nM in ZELHα cells. This sensitivity and different affinity of ERs for E2 is in perfect line with our previous studies using the same cell lines [18,19], which confirms the reproducibility and robustness of established cell models.

**Figure 2:**
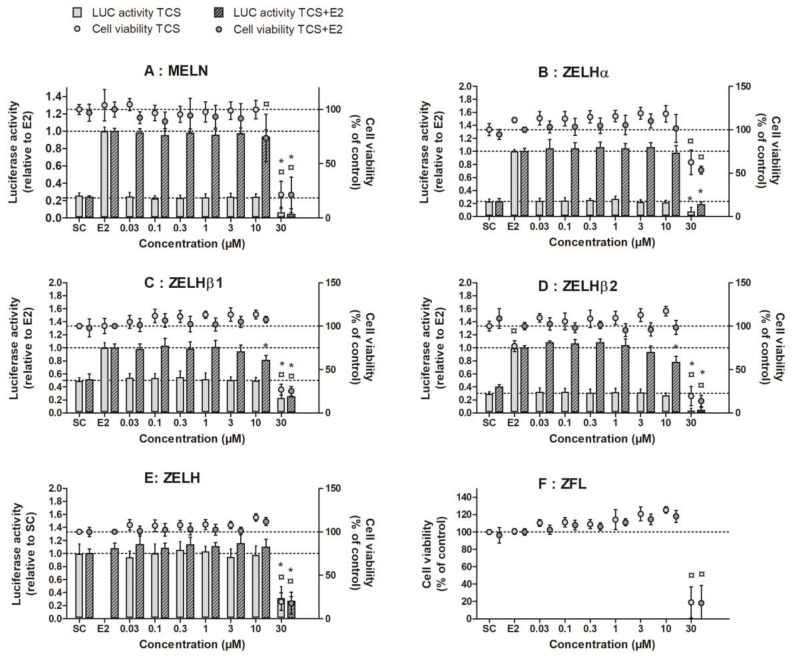
Luciferase response (LUC, bars) and cell viability (circles) of MELN (**A**), ZELHα **(B**), ZELHβ1 (**C**), ZELHβ2 (**D**), ZELH (**E**) and ZFL (**F**) cells after exposure to TCS (0.03 to 30 µM) for 24 h (MELN) or 72 h (ZELH-zfERs), in presence (dark symbols) or absence (light symbols) of E2. MELN, ZELHβ1 and ZELHβ2 cells were co-exposed to TCS + E2 0.1 nM, and ZELHα, ZELH and ZFL cells to TCS + E2 1 nM. Data represent the mean (+/− SD) of a minimum of 3 independent experiments using technical triplicates. Data were normalized to E2 positive control for luciferase activity. SC: solvent control ¤: response significantly different from control cell viability *: response significantly different from control luciferase activity (Mann-Whitney, *p* < 0.05).

Across all ER-expressing reporter cell lines, exposure to between 0.01 and 10 µM TCS did not induce any ER transactivation (Figure 2A–D, light bars). A slight decrease in luciferase activity was noted at 30 µM, where a cytotoxic event was also evidenced, as measured by the MTT test (Figure 2A–D, light circles). When cells were co-exposed to E2, E2-induced luciferase remained unaffected by the addition of up to 10 µM of TCS in the MELN and ZELHα cells (Figure 2A,B, dark bars), while a slight but significant decrease in E2-induced luciferase activity was observed in ZELHβ1 and ZELHβ2 cells at 10 µM (Figure 2C,D, dark bars). Above 10 µM TCS, luciferase activity significantly decreased in all the reporter cell lines, in both the presence and absence of E2. The ER-independent decrease of luciferase activity and cell viability at 30 µM TCS was confirmed in the parental ZFL and ZELH cell lines that do not express any functional zfER (Figure 2E,F). Altogether, our results suggest that TCS did not interact with ER in zebrafish liver cells, either as an ER agonist or an ER antagonist.

### 2.2. Effect of Triclosan on Brain Aromatase Expression Using the Cyp19a1b-GFP Transgenic Zebrafish Embryo Assay (EASZY Assay)

**Figure 3:**
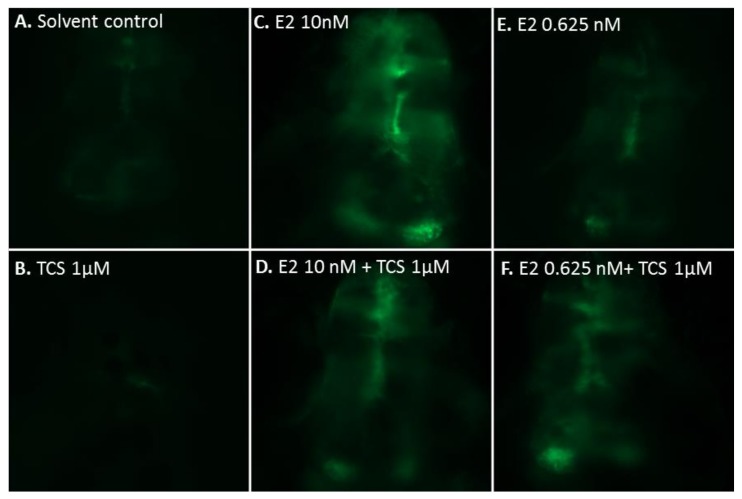
Dorsal view of the developing brain showing in vivo GFP expression (green signal) in the radial glial cells (RGCs) of transgenic cyp19a1b-GFP zebrafish (zf) embryos (4-dpf old) exposed to (**A**) solvent (DMSO); (**B**) TCS (1 µM); (**C**) and (**E**) E2 (10 nM and 0.625 nM); and (**D**,**F**) E2 + TCS for 96 h.

**Figure 4:**
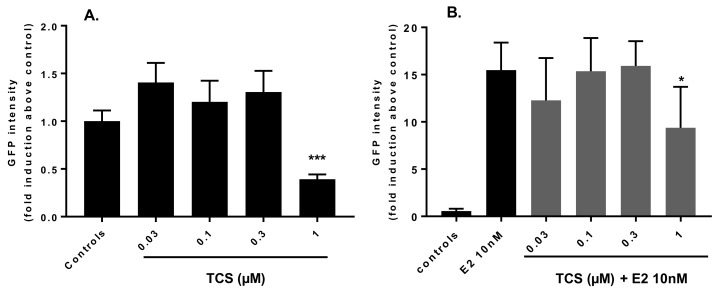
Effect of TCS on cyp19a1b expression in transgenic zebrafish larvae. Zebrafish embryos were exposed to TCS from 0.03 to 1 µM alone (**A**) or in co-exposure to E2 10 nM (**B**); from 3 h post fertilization (hpf) for 96 h with daily renewal of medium. GFP intensity was measured on day 4 on living organisms by fluorescence microscopy. Data are mean values ± SEM of a minimum of 2 experiments. *N* = 2 independent experiments with *n* = 20 embryos per condition per experiment. * and ***: significantly different from (**A**) control or (**B**) E2 groups (*p* < 0.05 and *p* < 0.001, respectively, Mann–Whitney test).

Representative patterns of GFP expression in the developing brain of transgenic cyp19a1b-GFP zebrafish embryos are shown in Figure 3. Typically, a low GFP signal is observed in the control fish (Figure 3A) while in the E2-treated fish, a strong GFP signal is observed in the radial glial cells (Figure 3C,E). To investigate the potential effects of TCS on the expression of ER-regulated genes, transgenic cyp19a1b-GFP zebrafish embryos were exposed to graded concentrations of TCS, either alone or in combination with E2, from 3 h post fertilization (hpf) to 96 hpf. The quantification of the GFP signal after chemical treatment is presented in Figure 4.

TCS alone did not induce any GFP expression in the EASZY assay (Figure 3A). In contrast, TCS 1 µM consistently and significantly reduced GFP intensity by 60% of the control group (*p* = 0.0002) (Figure 3B and Figure 4A). To further assess the interference of TCS with the ER-regulated expression of the cyp19a1b gene, zf embryos were exposed to different concentrations of TCS in the presence of E2 10 nM. At this concentration, E2 alone strongly induced GFP intensity by a factor of 15 as compared to controls (Figure 3C and Figure 4B). Co-exposure to graded concentrations of TCS had no significant effects on E2-induced cyp19a1b expression from 0.03 to 0.3 µM, but significantly decreased the E2-induced GFP intensity by 30% at 1 µM TCS (Figure 3D and Figure 4B, *p* = 0.034).

**Figure 5:**
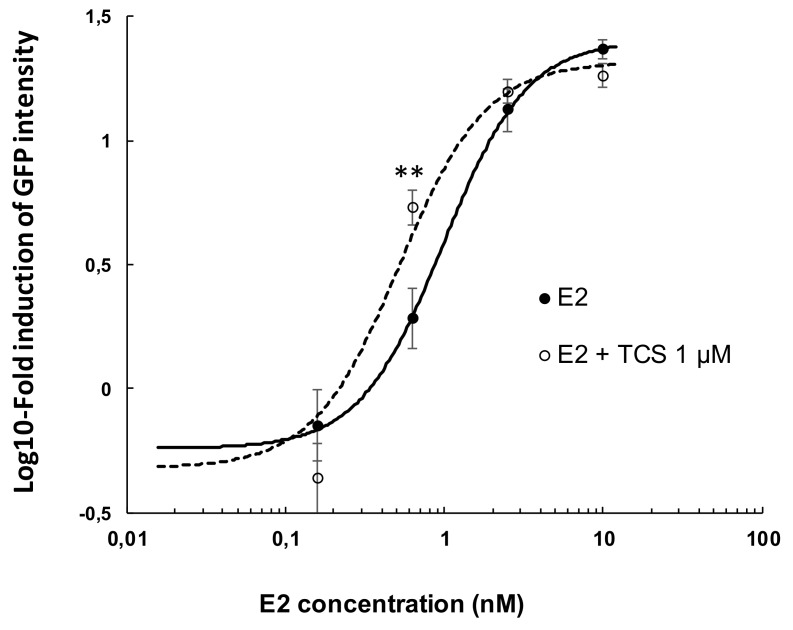
Effect of E2 from 0.16 to 10 nM alone (solid line) or in the presence of TCS 1 µM (dashed line) on cyp19a1b expression in transgenic zebrafish embryos. Data represent mean of log-10 transformed fold induction values ± SEM (**: significant difference at *p* < 0.01, Mann–Whitney test). *N* = 2 independent experiments with *n* = 20 embryos per condition per experiment.

We then further investigated the ability of TCS 1 µM to modulate the E2 concentration-dependent induction of GFP (Figure 5). E2 alone induced GFP in a concentration-dependent manner with an EC_50_ = 0.98 nM. In the presence of TCS 1 µM, modulation of the E2-induced response was observed, resulting in a reduction of the EC_50_ of E2 to 0.52 nM. This was the consequence of a significant 2.8-fold increase in GFP intensity at 0.625 nM E2 in the presence of TCS 1 µM, as compared to E2 alone (*p* = 0.0095) (Figure 3E,F). Furthermore, TCS reduced the response induced by E2 at 10 nM (*p* = 0.061) by 20%, a trend which confirmed the inhibitory effect of 1 µM TCS, as noted in Figure 4.

## 3. Discussion

The in vitro assay data in ZELHs and MELN reporter cell lines demonstrate the lack of agonistic and antagonistic activity of TCS toward zfERs and hERα, in the range of tested concentrations (0.03–30 µM as nominal concentrations). A small but significant decrease in E2-induced luciferase activity was observed at TCS 10 µM in ZELHβ1 and ZELHβ2 cells. However, the decrease was minimal, and occurred close to cytotoxic concentrations, suggesting an unspecific effect on luciferase activity rather than a direct effect mediated by zfERβs. There is a lot of information regarding the estrogenic and anti-estrogenic effects of TCS in vitro. The absence of ER transactivation by TCS alone in the zebrafish reporter cell lines is in accordance with previous studies based on human cells transfected with recombinant fish [16] or human ERα [13,14]. To our knowledge, no information on the implication of zfERβ1 and zfERβ2 subtypes in TCS responses is available so far, impeding any comparison. In contrast to the lack of estrogenic effects, the absence of anti-estrogenic activity observed in the zf and human reporter cell lines differs from the findings of previous reports. With the exception of one study, which showed no effect of TCS on 17α-ethynylestradiol (EE2)-induced hERα activation in T47D-kbLuc breast cancer cell line [22], several in vitro studies have described TCS as anti-estrogenic in transactivation and proliferative assays using human cells [12,13,14]. Differences between test systems, e.g., organism (fish vs. human), tissue (breast cancer cells vs. hepatocytes), and ER subtype (ERα vs. ERβ1/β2), may have contributed to the differences in observations.

In the present study, we noticed a decrease in reporter gene activity at 30 µM TCS irrespective of E2 in all the cell lines, which paralleled a marked decrease in cell viability. TCS was shown to induce a caspase-dependent apoptosis in neuronal cells [23,24], and to reduce cell viability by targeting cell proliferation in mouse embryonic stem cells [25] and MCF-7 cells [26]. Thus, the decreased cell viability observed in ZFL-derived cells and in MELN cells at 30 µM is in line with the broad cellular toxicity of TCS reported in the high µM range.

The (anti-)estrogenic effects of TCS were further investigated in vivo using transgenic zf embryos, which express GFP under the control of cyp19a1b promoter in RGCs [21]. Cyp19a1b codes for the brain aromatase (aromatase B)—it is responsible for the conversion of androgens into estrogens, and its expression is highly inducible by ER agonists at the embryonic stage, by way of an autoregulatory loop [27,28]. In the present study, we showed that TCS alone did not induce the expression of cyp19a1b. These results are in line with the in vitro data obtained in this study in the same model species.

At 1 µM, TCS alone significantly decreased GFP intensity in comparison to controls. The significantly decreased GFP observed in the presence of TCS alone is unlikely due to its acute toxic effect on embryos, as no significant mortality was observed under this exposure condition. Furthermore, among the more than 100 substances tested in the EASZY assay so far [17,21,29,30], none has been reported to decrease basal cyp19a1b expression in zf embryos, even those that are known to act as anti-estrogens, such as ICI 182,780. It is therefore unlikely that the observed decrease reflects TCS anti-estrogenic activity through direct interaction with ER, as also supported by our in vitro data.

TCS is known to target the thyroid axis in many organisms, including, in fish, at the larval and adult stages [31,32]. Thyroid hormones are essential to central nervous system development and function [33]. For instance, triiodothyronine (T3) is necessary for the differentiation of glial cells onto oligodendrocytes and astrocytes [34]. TCS was shown to alter the thyroid system of *Cyprinodon variegatus* larvae, including T3 levels [32]. Thus, we may hypothesize that the observed decrease in GFP intensity observed at 1 µM TCS in zf embryos may result from neuro-developmental toxicity, including effects on RGCs expressing cyp19a1b. Further investigation at the embryonic stage in fish would be warranted, in order to study the potential direct or indirect effects of TCS on RGCs, and its consequences for brain development.

Our study suggests that TCS acts in a complex manner in the presence of E2. On the one hand, TCS at 1 µM significantly potentiates the GFP expression induced by E2, but only at a low concentration (i.e., 0.625 nM), resulting in a shift of E2 EC50. On the other hand, TCS tends to decrease the effect of E2, but only at the highest E2 concentrations (i.e., 10 nM). The potentiation of EE2 effects by TCS has been observed in the uterotrophic assay following a 21-day exposure period, while TCS alone lacked estrogenic effect [35]. Overall, our results show that TCS can act on the ER-regulated brain aromatase as an ambivalent substance, since its effects appear to be dependent on exposure conditions and concentration ratios between E2 and TCS. Interestingly, our observations support recent data regarding adult medaka, where TCS exposure led to either the inhibition of VTG synthesis in males, or an increased VTG synthesis in reproductive active females [10]. Other studies of adult carp (*Cyprinus carpio*) reported the ability of a 42-day exposure to TCS to increase VTG levels through non-ER pathways [36,37]. This induction resulted from increased E2 synthesis in gonads, i.e., gonadal aromatase (Cyp19a1a) induction. The authors also noticed aromatase (Cyp19a1b) induction in the hypothalamus, likely due to an increase in plasmatic E2-levels following induction of gonadal aromatase. The hypothalamus-pituitary gonadal (HPG) axis is not functional at the investigated embryonic stage, impeding the assessment of such effects of TCS in the EASZY assay. Altogether, our data provide complementary information supporting the hypothesis that steroid levels may influence the endocrine-disrupting effects of TCS in mammalian and non-mammalian models, through non-ER mediated but still undefined mechanisms.

In summary, among the possible TCS toxicity pathways, our study addressed the very specific effect of TCS on zfER transactivation and the ER-regulated expression of brain aromatase in zebrafish reporter gene assays. Our results demonstrated that TCS lacks agonistic and antagonistic activities towards zfER α, β1 and β2 subtypes, and hERα in vitro. At the organism level, TCS interfered with the expression of the ER-regulated brain aromatase in an ambivalent manner in zebrafish, irrespective of direct binding to and transactivation of zfERs.

## 4. Materials and Methods

### 4.1. Chemicals and Reagents

17β-estradiol (E2, CAS No. 50-28-2) and triclosan (TCS, CAS No. 3380-34-5) were purchased from Sigma-Aldrich (Saint-Quentin Fallavier, France). Dimethylsulfoxide (DMSO), Leibovitz 15 culture medium (L-15), fetal calf serum (FCS), 4-(2-hydroxy-ethyl)-1-piperazineethanesulfonic acid (HEPES), epidermal growth factor (EGF), G418, 3-[4,5-dimethylthiazol-2-yl]-2,5-diphenyltetrazolium-bromide (MTT), and d-luciferin were purchased from Sigma Aldrich. Dulbecco’s Modified Eagle Medium High Glucose (DMEM HG) powder, F-12 nutrient mixture (Ham’s F12) powder, penicillin, and streptomycin were purchased from Gibco. Insulin, hygromycin B, and sodium bicarbonate were purchased from Dominique Dutscher (Issy-les-Moulineaux, France).

### 4.2. In Vitro Assays: Cell Culture, Luciferase and Cell Viability Assays

The zf in vitro assays were derived from the zf liver (ZFL) cell line, which was stably transfected, firstly by an ERE-driven luciferase gene, yielding the ZELH cell line, secondly by zfERα subtype, yielding the ZELH-zfERα, or by zfERβ1 subtype yielding the ZELH-zfERβ1 cell lines, or by zfERβ2 subtype yielding the ZELHβ2 cell lines. The establishment of these cell models, and their response to different classes of well-known xeno-estrogens has been previously described [18,19]. In addition, the ZFL and ZELH cell lines were used as ER-negative controls. In addition to the zebrafish cell models, we used the human-derived MELN cell line [20], kindly provided by Dr. Patrick Balaguer (INSERM, Montpellier, France). The MELN cells are derived from the MCF-7 cells, which endogenously express the hERα, but no functional hERβ (P. Balaguer, personal communication).

Conditions for routine cell culture and exposure to chemicals have been detailed previously [19,20]. Briefly, ZELH-zfERs cells were seeded in 96-well white opaque culture plates (Greiner CellStar™, Dominique Dutscher, Brumath, France) at 25,000 cells per well in phenol red free LDF-DCC medium (containing 50% of L-15, 35% of DMEM HG, 15% of Ham’s F12, 15 mM of HEPES, 0.15 g/L of sodium bicarbonate, 0.01 mg/mL of insulin, 50 ng/mL of EGF, 50 U/mL of penicillin and streptomycin antibiotics, and 5% *v*/*v* stripped FCS). MELN were seeded at 80,000 cells per well cell line in steroid-free DMEM medium. Cells were left to adhere for 24 h. Following this, they were exposed in triplicates to serial dilutions of test compound for either 72 h at 28 °C for zebrafish cells, or 16 h at 37 °C for MELN cells. After exposure, luciferase activity was measured. The medium was removed and replaced by 50 μL per well of medium containing 0.3 mM luciferin. The luminescence signal was measured in living cells using a microtiter plate luminometer (Synergy H4, BioTek Instruments, Luzern, Switzerland). The effect of test chemicals on cell viability was assessed by using the 3-(4,5-dimethyl-thiazol-2-yl)-2,5-diphenyltetrazolium bromide (MTT) assay [38]. After cell exposure, culture medium was removed and replaced by 100 μL of medium containing 0.5 mg/mL MTT. Cells were incubated for 3 h. In metabolically active cells, MTT was reduced onto a blue formazan precipitate, which was dissolved by adding 100 μL of DMSO after removal of MTT containing medium. Plates were then read at 570 nm against a 640 nm reference wavelength on a microplate reader (KC-4, BioTek Instruments, Colmar, France), and results were expressed as absorbance relative to control cells.

### 4.3. In Vivo Zebrafish Bioassays

In vivo anti-estrogenicity of TCS was assessed using the transgenic cyp19a1b-GFP zebrafish line, previously developed [39] and well characterized with different classes of estrogens and xeno-estrogen compounds [21]. The assay procedure for individual chemical testing has been described in detail by Brion et al. [21]. To briefly summarize, 20 fertilized transgenic eggs were selected for each experimental group and exposed for 96 h in 25 mL of acclimated water in glass crystallizers. Serial dilutions were tested with a final volume of solvent (DMSO) of 0.01% *v*/*v*, a concentration without any effects on embryo development or GFP expression. In each experimental series, positive (EE2 0.05 nM) and DMSO controls were included as separate experimental groups. Exposed embryos were incubated at 28 °C, under semi-static conditions with complete renewal of the medium daily. After the exposure period, each zebrafish larva was photographed using a Zeiss Axio Imager.Z1 microscope equipped with an AxioCam Mrm camera (Zeiss GmbH, Gottingen, Germany) to measure GFP expression in the brain. Image analysis was performed using the ImageJ software, and fluorescence data was treated exactly as previously described [21].

Animal maintenance was performed under strict respect of animal welfare. The assays based on zebrafish embryos used in this study are not subjected to animal experiments according to the European Directive 2010/63/EU, and are to be considered as alternative methods for animal experiments.

### 4.4. Data Analysis

Dose-response curves were fitted to the experimental data using the Hill equation as provided in the RegTox 7.5 Microsoft Excel™ macro [40]. Significant effects on luciferase induction, GFP intensity, and cell viability in exposed conditions were determined by comparing each condition to solvent control (estrogenic effects), or to E2 control (antiestrogenic effects), applying a non-parametric bilateral Mann–Whitney statistical test with α set at 5%. Furthermore, DMSO and water controls of zebrafish cyp19ab-GFP larvae were pooled together when no significant differences were observed, applying a non-parametric bilateral Mann–Whitney statistical test with α set at 5%.

## References

[1] SCCS (Scientific Committee on Consumer Safety) (2010). Opinion on Triclosan (Antimicrobial Resistance).

[2] Dann A.B., Hontela A. (2011). Triclosan: Environmental exposure, toxicity and mechanisms of action. J. Appl. Toxicol..

[3] Loos R., Carvalho R., Antonio D.C., Cornero S., Locoro G., Tavazzi S., Paracchini B., Ghiani M., Lettieri T., Blaha L. (2013). EU-wide monitoring survey on emerging polar organic contaminants in wastewater treatment plant effluents. Water Res..

[4] Brausch J.M., Rand G.M. (2011). A review of personal care products in the aquatic environment: Environmental concentrations and toxicity. Chemosphere.

[5] Kolpin D.W., Furlong E.T., Meyer M.T., Thurman E.M., Zaugg S.D., Barber L.B., Buxton H.T. (2002). Pharmaceuticals, hormones, and other organic wastewater contaminants in US streams, 1999–2000: A national reconnaissance. Environ. Sci. Technol..

[6] Schultz M.M., Bartell S.E., Schoenfuss H.L. (2012). Effects of Triclosan and Triclocarban, Two Ubiquitous Environmental Contaminants, on Anatomy, Physiology, and Behavior of the Fathead Minnow (*Pimephales promelas*). Arch. Environ. Contam. Toxicol..

[7] Foran C.M., Bennett E.R., Benson W.H. (2000). Developmental evaluation of a potential non-steroidal estrogen: Triclosan. Mar. Environ. Res..

[8] Raut S.A., Angus R.A. (2010). Triclosan has endocrine-disrupting effects in male western mosquitofish, *Gambusia affinis*. Environ. Toxicol. Chem..

[9] Ishibashi H., Matsumura N., Hirano M., Matsuoka M., Shiratsuchi H., Ishibashi Y., Takao Y., Arizono K. (2004). Effects of triclosan on the early life stages and reproduction of medaka Oryzias latipes and induction of hepatic vitellogenin. Aquat. Toxicol..

[10] Horie Y., Yamagishi T., Takahashi H., Iguchi T., Tatarazako N. (2018). Effects of triclosan on Japanese medaka (*Oryzias latipes*) during embryo development, early life stage and reproduction. J. Appl. Toxicol..

[11] Huang H.Y., Du G.Z., Zhang W., Hu J.L., Wu D., Song L., Xia Y.K., Wang X.R. (2014). The in vitro estrogenic activities of triclosan and triclocarban. J. Appl. Toxicol..

[12] Henry N.D., Fair P.A. (2013). Comparison of in vitro cytotoxicity, estrogenicity and anti-estrogenicity of triclosan, perfluorooctane sulfonate and perfluorooctanoic acid. J. Appl. Toxicol..

[13] Ahn K.C., Zhao B., Chen J., Cherednichenko G., Sanmarti E., Denison M.S., Lasley B., Pessah I.N., Kultz D., Chang D.P.Y. (2008). In vitro biologic activities of the antimicrobials triclocarban, its analogs, and triclosan in bioassay screens: Receptor-based bioassay screens. Environ. Health Perspect..

[14] Gee R.H., Charles A., Taylor N., Darbre P.D. (2008). Oestrogenic and androgenic activity of triclosan in breast cancer cells. J. Appl. Toxicol..

[15] Tarnow P., Tralau T., Hunecke D., Luch A. (2013). Effects of triclocarban on the transcription of estrogen, androgen and aryl hydrocarbon receptor responsive genes in human breast cancer cells. Toxicol. Vitro.

[16] Miyagawa S., Lange A., Hirakawa I., Tohyama S., Ogino Y., Mizutani T., Kagami Y., Kusano T., Ihara M., Tanaka H. (2014). Differing Species Responsiveness of Estrogenic Contaminants in Fish Is Conferred by the Ligand Binding Domain of the Estrogen Receptor. Environ. Sci. Technol..

[17] Le Fol V., Ait-Aissa S., Sonavane M., Porcher J.M., Balaguer P., Cravedi J.P., Zalko D., Brion F. (2017). In vitro and in vivo estrogenic activity of BPA, BPF and BPS in zebrafish-specific assays. Ecotoxicol. Environ. Saf..

[18] Sonavane M., Creusot N., Maillot-Marechal E., Pery A., Brion F., Ait-Aissa S. (2016). Zebrafish-based reporter gene assays reveal different estrogenic activities in river waters compared to a conventional human-derived assay. Sci. Total Environ..

[19] Cosnefroy A., Brion F., Maillot-Marechal E., Porcher J.M., Pakdel F., Balaguer P., Ait-Aissa S. (2012). Selective Activation of Zebrafish Estrogen Receptor Subtypes by Chemicals by Using Stable Reporter Gene Assay Developed in a Zebrafish Liver Cell Line. Toxicol. Sci..

[20] Balaguer P., Francois F., Comunale F., Fenet H., Boussioux A.M., Pons M., Nicolas J.C., Casellas C. (1999). Reporter cell lines to study the estrogenic effects of xenoestrogens. Sci. Total Environ..

[21] Brion F., Le Page Y., Piccini B., Cardoso O., Tong S.K., Chung B.C., Kah O. (2012). Screening Estrogenic Activities of Chemicals or Mixtures In vivo Using Transgenic (*cyp19a1b*-GFP) Zebrafish Embryos. PLoS ONE.

[22] Louis G.W., Hallinger D.R., Stoker T.E. (2013). The effect of triclosan on the uterotrophic response to extended doses of ethinyl estradiol in the weanling rat. Reprod. Toxicol..

[23] Park B.K., Gonzales E.L.T., Yang S.M., Bang M.J., Choi C.S., Shin C.Y. (2016). Effects of Triclosan on Neural Stem Cell Viability and Survival. Biomol. Ther..

[24] Szychowski K.A., Sitarz A.M., Wojtowicz A.K. (2015). Triclosan induces fas receptor-dependent apoptosis in mouse neocrotical neurons in vitro. Neuroscience.

[25] Chen X.J., Xu B., Han X.M., Mao Z.L., Chen M.J., Du G.Z., Talbot P., Wang X.R., Xia Y.K. (2015). The effects of triclosan on pluripotency factors and development of mouse embryonic stem cells and zebrafish. Arch. Toxicol..

[26] Liu B.Q., Wang Y.Q., Fillgrove K.L., Anderson V.E. (2002). Triclosan inhibits enoyl-reductase of type I fatty acid synthase in vitro and is cytotoxic to MCF-7 and SKBr-3 breast cancer cells. Cancer Chemother. Pharmacol..

[27] Le Page Y., Menuet A., Kah O., Pakdel F. (2008). Characterization of a cis-acting element involved in cell-specific expression of the zebrafish brain aromatase gene. Mol. Reprod. Dev..

[28] Menuet A., Pellegrini E., Brion F., Gueguen M.M., Anglade I., Pakdel F., Kah O. (2005). Expression and estrogen-dependent regulation of the zebrafish brain aromatase gene. J. Comp. Neurol..

[29] Cano-Nicolau J., Garoche C., Hinfray N., Pellegrini E., Boujrad N., Pakdel F., Kah O., Brion F. (2016). Several synthetic progestins disrupt the glial cell specific-brain aromatase expression in developing zebra fish. Toxicol. Appl. Pharmacol..

[30] Neale P.A., Altenburger R., Ait-Aissa S., Brion F., Busch W., Umbuzeiro G.d.A., Denison M.S., Du Pasquier D., Hilscherova K., Hollert H. (2017). Development of a bioanalytical test battery for water quality monitoring: Fingerprinting identified micropollutants and their Contribution to effects in surface water. Water Res..

[31] Pinto P.I.S., Guerreiro E.M., Power D.M. (2013). Triclosan interferes with the thyroid axis in the zebrafish (*Danio rerio*). Toxicol. Res..

[32] Schnitzler J.G., Frederich B., Dussenne M., Klaren P.H.M., Silvestre F., Das K. (2016). Triclosan exposure results in alterations of thyroid hormone status and retarded early development and metamorphosis in *Cyprinodon variegatus*. Aquat. Toxicol..

[33] Di Liegro I. (2008). Thyroid hormones and the central nervous system of mammals (Review). Mol. Med. Rep..

[34] Noda M. (2015). Possible role of glial cells in the relationship between thyroid dysfunction and mental disorders. Front. Cell. Neurosci..

[35] Stoker T.E., Gibson E.K., Zorrilla L.M. (2010). Triclosan Exposure Modulates Estrogen-Dependent Responses in the Female Wistar Rat. Toxicol. Sci..

[36] Wang F., Guo X., Chen W., Sun Y., Fan C. (2017). Effects of triclosan on hormones and reproductive axis in female Yellow River carp (*Cyprinus carpio*): Potential mechanisms underlying estrogen effect. Toxicol. Appl. Pharmacol..

[37] Wang F., Liu F., Chen W., Xu R., Wang W. (2018). Effects of triclosan (TCS) on hormonal balance and genes of hypothalamus-pituitary-gonad axis of juvenile male Yellow River carp (*Cyprinus carpio*). Chemosphere.

[38] Mosmann T. (1983). Rapid colorimetric assay for cellular growth and survival —Application to proliferation and cyto-toxicity assays. J. Immunol. Methods.

[39] Tong S.K., Mouriec K., Kuo M.W., Pellegrini E., Gueguen M.M., Brion F., Kah O., Chung B.C. (2009). A *cyp19a1b*-GFP (Aromatase B) Transgenic Zebrafish Line That Expresses GFP in Radial Glial Cells. Genesis.

[40] Vindimian E. REGTOX: Macro Excel^TM^ Pour Dose-Réponse. http://www.normalesup.org/~vindimian/fr_index.html.

